# Texture-Based Classification to Overcome Uncertainty between COVID-19 and Viral Pneumonia Using Machine Learning and Deep Learning Techniques

**DOI:** 10.3390/diagnostics14101017

**Published:** 2024-05-15

**Authors:** Omar Farghaly, Priya Deshpande

**Affiliations:** Data-Intensive Computing Distributed Systems Laboratory, Department of Electrical and Computer Engineering, Marquette University, Milwaukee, WI 53233, USA

**Keywords:** texture-based features, classification, machine learning, deep learning, COVID-19, viral pneumonia

## Abstract

The SARS-CoV-2 virus, responsible for COVID-19, often manifests symptoms akin to viral pneumonia, complicating early detection and potentially leading to severe COVID pneumonia and long-term effects. Particularly affecting young individuals, the elderly, and those with weakened immune systems, the accurate classification of COVID-19 poses challenges, especially with highly dimensional image data. Past studies have faced limitations due to simplistic algorithms and small, biased datasets, yielding inaccurate results. In response, our study introduces a novel classification model that integrates advanced texture feature extraction methods, including GLCM, GLDM, and wavelet transform, within a deep learning framework. This innovative approach enables the effective classification of chest X-ray images into normal, COVID-19, and viral pneumonia categories, overcoming the limitations encountered in previous studies. Leveraging the unique textures inherent to each dataset class, our model achieves superior classification performance, even amidst the complexity and diversity of the data. Moreover, we present comprehensive numerical findings demonstrating the superiority of our approach over traditional methods. The numerical results highlight the accuracy (random forest (RF): 0.85; SVM (support vector machine): 0.70; deep learning neural network (DLNN): 0.92), recall (RF: 0.85, SVM: 0.74, DLNN: 0.93), precision (RF: 0.86, SVM: 0.71, DLNN: 0.87), and F1-Score (RF: 0.86, SVM: 0.72, DLNN: 0.89) of our proposed model. Our study represents a significant advancement in AI-based diagnostic systems for COVID-19 and pneumonia, promising improved patient outcomes and healthcare management strategies.

## 1. Introduction

The COVID-19 pandemic, originating as a respiratory virus in late 2019 in China, swiftly escalated into a global crisis, prompting the World Health Organization (WHO) to declare it a pandemic due to its rapid transmission and severe symptoms [1,2]. Its unprecedented spread has precipitated significant economic challenges and profound human suffering worldwide since its onset [1]. Governments worldwide have enacted stringent measures and embraced new lifestyles to mitigate its spread [3]. However, despite these efforts, the virus has undergone multiple mutations, rendering it more resistant to advanced treatment techniques [1], implying its persistence for an extended period.

Detecting and distinguishing COVID-19 from other causes of viral pneumonia early on is crucial, as it may progress to COVID pneumonia, a severe complication. However, COVID-19 shares most symptoms with other viral pneumonias like the flu, making symptom-based identification challenging. COVID pneumonia progresses slowly through the lungs, relying on the immune system for elimination, causing extensive damage and leading to long-term illness. In contrast, other pneumonias cause acute illnesses, with all symptoms appearing at once but not lasting as long. Symptom variability adds complexity to diagnosis; COVID-19 symptoms range from mild flu-like manifestations to severe respiratory distress, with some individuals showing no symptoms at all [4]. This similarity in symptoms often leads to misdiagnosis or delays in diagnosis. Initially, RT-PCR test kits were widely used for COVID-19 detection, but medical imaging modalities such as chest X-ray (CXR) and computed tomography (CT) have emerged as crucial alternatives. CXR and CT are popular for diagnosing lung anomalies, including COVID-19. Moreover, the accuracy of testing methods poses another challenge. While various techniques, such as PCR tests and antigen tests, are employed for COVID-19 detection, they differ in sensitivity and specificity. False-negative results are a concern, particularly when viral loads are low or if sample collection processes are suboptimal. These challenges underscore the need for comprehensive testing strategies and ongoing advancements in diagnostic techniques to effectively combat the spread of the virus [5].

In this study, we focus solely on CXR images due to their widespread availability and ease of acquisition. However, we acknowledge that other imaging modalities like CT and magnetic resonance imaging (MRI) also play vital roles in diagnosing respiratory diseases and could be integrated into future research endeavors.

Our primary novelty lies in the integration of advanced texture feature extraction techniques, including grey level co-occurrence matrix (GLCM), grey level difference method (GLDM), and wavelet-based texture analysis, within a DLNN model for the classification of lung images into COVID-19 and viral pneumonia categories. While previous studies have utilized deep learning models for medical image classification, such as [6,7,8], the incorporation of GLCM, GLDM, and wavelet texture features represents a novel approach to feature representation in this context. These texture features provide valuable information about the spatial relationships, gray-level variations, and frequency characteristics within the lung images, enabling the DLNN model to learn discriminative patterns for accurate disease classification. By harnessing the complementary strengths of texture analysis and deep learning, our methodology offers a novel framework for enhancing the accuracy and robustness of COVID-19 diagnosis from medical imaging data. This innovative approach contributes to the advancement of medical image analysis techniques and holds significant potential for improving clinical decision-making and patient outcomes in the context of respiratory disease diagnosis.

Furthermore, our study proposes an integrated approach utilizing RF, SVM, and DLNN techniques for classification. The choice of these classifiers is grounded in both empirical evidence and theoretical insights. RF harnesses the collective predictive power of multiple decision trees, making it well-suited for handling data complexity and noise commonly encountered in medical imaging datasets. SVM excels at delineating optimal decision boundaries between classes, facilitating precise segregation of data points in high-dimensional feature spaces. DLNN, a groundbreaking classification paradigm capable of autonomously discerning intricate features from data, enhances classification precision. Moreover, empirical evidence from previous studies has demonstrated the efficacy of texture analysis techniques and classifiers in various medical imaging applications, including the diagnosis of lung diseases such as pneumonia and lung cancer. By combining these established techniques with state-of-the-art classifiers, we hypothesize that our proposed method will outperform existing models in the classification of lung images into COVID-19 and viral pneumonia categories. Through rigorous experimentation and comparative analysis, as detailed in Section 4, we aim to validate this hypothesis and provide empirical evidence supporting the superiority of our proposed method. By elucidating the theoretical basis and empirical rationale behind our approach, we aim to ensure transparency and rigor in our methodology, thereby strengthening the scientific foundation of our research.

Our contribution extends beyond mere classification; we present a comprehensive evaluation of our model’s performance using metrics such as categorical accuracy, confusion matrix, precision, recall, F1-Score, and ROC. Additionally, we provide insights into the rationale behind our model’s design choices, offering a robust framework for accurate chest X-ray image categorization.

In addition, Section 4 of our paper provides an exhaustive comparative analysis of existing literature. This section critically evaluates and compares our integrated approach with previous studies, elucidating the advancements and contributions of our methodology. Through this comparative analysis, our objective is to underscore the novelty and efficacy of our approach in the domain of COVID-19 diagnosis utilizing medical imaging data.

In summary, our study pioneers a novel approach combining texture-based feature extraction with advanced classification techniques for accurate COVID-19 and pneumonia differentiation from CXR images. Our findings not only advance the field of medical imaging but also hold significant promise for improving diagnostic accuracy and patient care in respiratory medicine.

The present study is structured as follows: Section 2 provides a detailed discussion of the previous state-of-the-art related work that has been conducted recently. In Section 3, we elucidate the implementation of our system and the design choices we made, along with the evaluation methodology adopted in this study. The evaluation results are presented and described in Section 4. Furthermore, Section 5 provides a comprehensive discussion of the model employed in this study. In Section 6, we present our research work conclusions, followed by a discussion of the future directions in Section 7.

## 2. Related Work

This section presents a comprehensive review of recent state-of-the-art studies focusing on the utilization of CT or CXR images in DLNN models for COVID-19 detection and diagnosis. Our objective is to critically examine these studies, highlighting their methodologies, strengths, limitations, and relevance to the proposed research.

Aslan et al. [2] employed pre-trained convolutional neural network (CNN) models for COVID-19 classification from CXR images, preceded by lung segmentation as a pre-processing step. This segmentation technique, achieved through an artificial neural network (ANN), aims to eliminate irrelevant information and improve model accuracy. While their approach yielded high accuracy ranging from 95.05% to 96.29%, it heavily relied on manual segmentation, which may limit scalability and automation. In contrast, Khan et al. [3] implemented DL models without prior segmentation of CXR images, achieving comparable accuracy rates ranging from 93.9% to 96%. However, the absence of segmentation may introduce noise and irrelevant information, potentially affecting model performance. Additionally, ref. [9] introduced RADIC, a diagnostic tool combining deep learning and quad-radiomics for COVID-19 diagnosis from chest CT and X-ray scans. While promising, challenges related to feature selection and model interpretability were noted, underscoring the need for further validation in clinical settings.

Similarly, ref. [10] proposed cascaded DL classifiers for computer-aided diagnosis of COVID-19 and pneumonia from X-ray scans, enhancing feature extraction through hierarchical architectures. However, scalability issues and computational resource requirements may limit its practical applicability. CoroNet [11], a deep neural network tailored for COVID-19 detection from CXR images, showcased promising diagnostic capabilities. Yet, concerns regarding dataset diversity and image quality were raised, necessitating further validation studies. Moreover, ref. [12] introduced a wavelet-based DL pipeline for COVID-19 diagnosis using CT slices, leveraging multi-resolution information. However, computational complexity and scalability issues remain challenges for its implementation. Similarly, ref. [13] proposed a texture-based radiomics analysis framework for coronavirus diagnosis, emphasizing feature extraction from medical images. Yet, concerns regarding feature selection and dataset heterogeneity were highlighted. Furthermore, ref. [14] introduced a fusion model combining handcrafted and DL features for COVID-19 diagnosis, aiming to capture complementary information from CXR images. However, the model’s performance may depend on feature selection and integration.

While ref. [15] achieved high accuracy in X-ray image detection of pneumonia and COVID-19 patients, concerns about dataset bias, model generalization, and interpretability were noted, emphasizing the need for robust validation. Similarly, ref. [16] achieved high accuracy in classifying COVID-19 cases from CXR images, yet challenges related to dataset bias and model generalizability were acknowledged, necessitating further investigation. Moreover, studies by [17,18,19] introduced various DL approaches for COVID-19 detection from medical images, each with distinct methodologies and performance metrics.

While previous studies have made significant strides in COVID-19 diagnosis using DLNN techniques, our proposed research aims to address several key gaps and existing limitations. For instance, concerns regarding dataset bias, model generalizability, and interpretability have been noted across various studies.

Specifically, imbalanced datasets used in some studies, such as those by Aslan et al. [2] and Khan et al. [3], may lead to biased model predictions due to the disproportionate representation of COVID-19 cases. Additionally, uncertainties regarding model generalizability across different healthcare settings, as highlighted in the study by [20], can limit the applicability of DL models in diverse clinical environments. Moreover, the lack of interpretability in DL models can hinder their adoption by healthcare professionals, who may be hesitant to rely solely on model predictions without clear insights into the underlying decision-making process.

In our proposed research, we aim to mitigate these limitations by integrating advanced texture feature extraction techniques with DL models, offering a richer representation of image data. By combining these techniques, we seek to enhance diagnostic accuracy and robustness, addressing the challenges encountered in previous studies. Additionally, our study provides a comprehensive evaluation framework and discusses the rationale behind the proposed methodology, contributing to the advancement of COVID-19 diagnosis from medical imaging data.

## 3. Methodology

In this section, we will discuss the methodology utilized in this project. Algorithm 1 outlines the steps taken in this study.
**Algorithm 1** The algorithm for the proposed model**for** i in range (0,len(dataset_Images) **do**// Pre-processing stepsImage resize;Adaptive histogram equalization;Normalization;// Extracting Texture Features from images**end for****while** Feature_pool≠0 **do**    TF←cat(Feature_pool)    X←FisherScore(TF)**end while****for** i in range (0,len(X) **do**    Model_classification=Classification(inputs=[X])    Model_classification.fit()    LossCalculator    OptimizeParameter**end for**

### 3.1. Dataset

First, we begin with the CXR image dataset acquired from Kaggle [21], which originally consisted of 10,192 normal, 3616 COVID-19 positive cases, and 1345 viral pneumonia images. This dataset was compiled by a team of researchers from Qatar University, Doha, Qatar, and the University of Dhaka, Bangladesh, along with their collaborators from Pakistan and Malaysia, in collaboration with medical doctors, creating a comprehensive database for AI-based diagnostic research. All the images are in portable network graphics (PNG) file format with a resolution of 299 × 299 pixels and are in 8-bit grayscale mode, providing 256 levels of gray.

**Figure 1:**
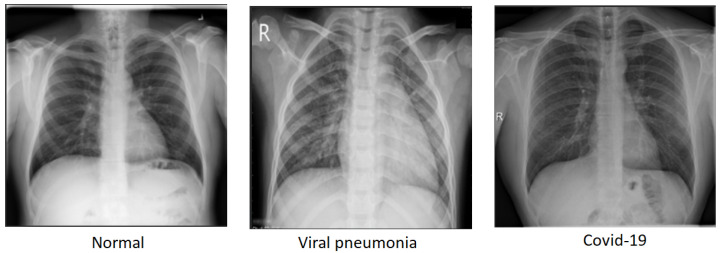
Snippet of the dataset.

To maintain a balanced dataset, we chose 1345 images randomly from each class, totaling 4035 images across the three classes. This equilibrium is vital for machine learning and computer vision tasks as it avoids biases towards classes with more samples and boosts the model’s ability to generalize to new data. With an even distribution of images per class, the model becomes adept at recognizing distinct features from all classes, not just the most prevalent ones. To address potential biases and ensure representativeness in our dataset, we took several deliberate steps to robustly manage our randomization process. We implemented a systematic random selection method that adheres to a uniform distribution, ensuring fairness by selecting images evenly across the dataset and preventing overrepresentation or underrepresentation of any specific category. By setting a random seed at the start, we guarantee reproducibility, allowing the random selection to be easily verified and replicated. Although we showcased just one output in the manuscript, we conducted the randomization process several times, yet the outcome consistently aligned. This uniformity across numerous randomizations bolsters the resilience of our method and indicates that the chosen images accurately represent the entire dataset. This approach, combined with our rigorous randomization methods, aimed to provide a comprehensive and unbiased representation of the dataset. In doing so, we enhanced its overall representativeness and strengthened the performance of the trained model. The COVID and normal class images were selected randomly from the original dataset. The train-test split was done using a ratio of 80% for the training set and 20% for the test set, as shown in Table 1. A snippet of the dataset is shown in Figure 1.

### 3.2. Feature Extraction Methods

**Figure 2:**
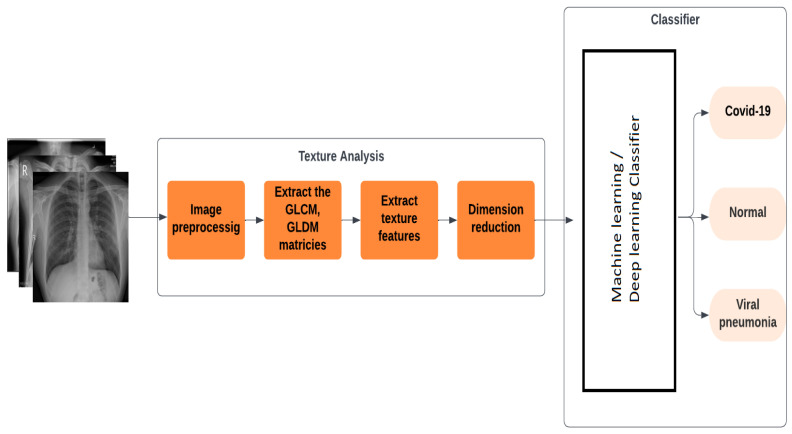
Architecture of the Proposed Methodology.

The images underwent a series of meticulous pre-processing steps to optimize their quality and prepare them for subsequent analysis within the deep learning framework. Initially, each image was resized to adhere to a standardized dimension of 299 × 299 pixels, ensuring consistency across the dataset and facilitating uniform processing by the deep learning algorithms. Resizing is applied only to images that do not match this standard dimension. This resizing serves as a precautionary measure to standardize the image dimensions and promote generalization across the dataset. Ensuring consistent image dimensions is crucial for machine learning models, as they require uniform input sizes for effective training and inference. Following resizing, histogram equalization was applied to enhance contrast and brightness levels within the images. This technique adjusts the distribution of pixel intensities to create a more balanced histogram, thereby improving the visibility of critical features and details in the images. Subsequently, we normalized the pixel values of the images to a standard range between 0 and 1 to ensure consistency across the dataset. By standardizing the numerical values of the pixels, normalization ensures stable and efficient training of the deep learning models, mitigating issues such as gradient vanishing or explosion. Collectively, these pre-processing steps play a vital role in optimizing the quality and uniformity of the image data, laying a solid foundation for accurate and reliable analysis within the deep learning framework, which is the first block in the block diagram shown in Figure 2.

While previous studies have utilized texture-based features for COVID-19 diagnosis, the novelty of our approach lies in the integration of GLCM and GLDM matrices with feature dimension reduction techniques. These methods offer a nuanced understanding of the spatial relationships, gray-level variations, and frequency characteristics present within the images. Such detailed information is crucial for effectively discerning the subtle differences between COVID-19 manifestations and other respiratory conditions.

The feature extraction methods, GLCM and GLDM, were chosen for their ability to capture intricate textural details from CXR images. These methods provide insights into the spatial relationships, gray-level variations, and frequency characteristics present within the images, essential for distinguishing between COVID-19 and other respiratory conditions.

Despite the prevalence of deep learning models in medical image analysis, we opted not to use them for feature extraction in this study. The decision was made due to the complexity of deep learning models, which often require large amounts of labeled data for training and can be computationally intensive. Given the challenges associated with obtaining labeled medical imaging data, especially in the context of rare diseases like COVID-19, and the computational resources required for training deep learning models, we sought alternative methods.

Instead, by integrating GLCM and GLDM matrices with feature dimension reduction techniques, we aimed to achieve two key objectives. Firstly, these methods enable us to extract a comprehensive set of discriminative features from the CXR images, capturing intricate nuances indicative of COVID-19 infection. Secondly, the feature dimension reduction techniques streamline the extracted feature set, enhancing computational efficiency and minimizing the risk of overfitting.

The synergy between advanced texture analysis methods and neural network classifiers is pivotal for improving the accuracy and robustness of COVID-19 classification. By feeding the neural network with the discriminative patterns derived from GLCM and GLDM matrices, we empower the model to learn and discern subtle distinctions between COVID-19 and other respiratory ailments more effectively. This integration facilitates a more precise and reliable diagnosis, ultimately contributing to enhanced patient care and clinical decision-making in the realm of respiratory medicine.

We skipped pre-trained DL models for feature extraction due to the unique complexities of medical imaging for COVID-19 diagnosis. Fine-tuning them demands significant computational resources and labeled medical data, scarce for rare diseases like COVID-19. Instead, we used advanced texture analysis methods like GLCM and GLDM, coupled with feature dimension reduction. This approach directly captures discriminative features from CXR images, reflecting COVID-19 pneumonia patterns while minimizing data labeling and computational needs. Our goal was to create a precise diagnostic tool tailored to COVID-19’s imaging challenges.

So the next step is to extract the GLCM and GLDM matrices and, from them, extract the texture features, which are combined in one pool to improve classification accuracy. To enhance runtime efficiency, we use the fisher score as a feature dimension reduction technique to select the most relevant feature vectors from the extraction process, as shown in the fourth block in Figure 2. Finally, these features are input into a neural network classifier to categorize images into normal, COVID, or viral pneumonia classes. The performance of the proposed method is assessed using the categorical accuracy metric.

**Figure 3:**
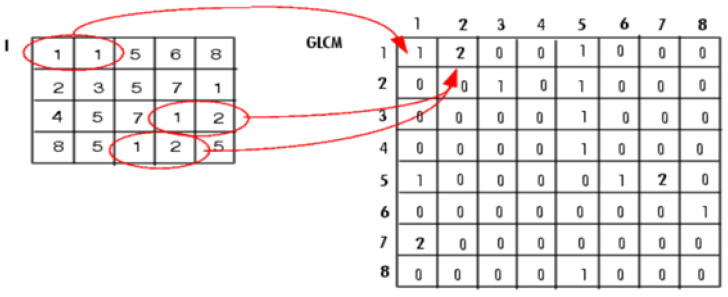
Example of constructing GLCM matrix [23].

GLCM is a texture feature extraction technique introduced by Haralick, Shanmugam, and Dinstein [22] that characterizes the texture of images by determining the spatial relationship of a pixel with a specific value. The GLCM is represented as a matrix that shows the likelihood of a particular gray level occurring in the neighborhood of any other gray level within a certain distance and angle [22]. The distance is expressed in pixels, while the angle is represented in degrees with four directions: 0°, 45°, 90°, and 135°. Figure 3 shows an example of constructing a GLCM matrix of distance one and direction 0°.

To calculate the GLCM texture features, Haralick, Shanmugam, and Dinstein [22] defined the features, including autocorrelation, contrast, correlation, dissimilarity, energy, entropy, homogeneity, and others, using equations.

In this study, we used energy, contrast, mean, max, min, standard deviation, area, correlation, dissimilarity, and homogeneity as texture features. Energy is the amount of varying gray intensity in the image, while contrast is the difference in the level of color or grayscale that appears in an image. The value of contrast will be zero if the neighboring pixels have the same amount. Correlation is the linear relationship between the degree of gray image. Dissimilarity measures variations in intensity levels between neighboring pixels and is considered high if the local region has high contrast. Homogeneity is the measure of distribution between neighboring pixels. The formulas for each feature are as follows:

(1) $$Energy=\sum_{i,j}P_{d}^{2}\left(i,j\right)$$

where $P_{d}\left(i,j\right)$ is a GLCM matrix of images with gray values *i* and *j*.

(2) $$Contrast=\sum_{i,j}{\left(i-j\right)}^{2}P_{d}\left(i,j\right)$$

(3) $$Correlation=\frac{\Sigma_{i,j}\left(i-u_{x}\right)\left(j-u_{y}\right)P_{d}\left(i,j\right)}{\sigma_{x}\sigma_{y}}$$

where $u_{x}$, $u_{y}$ is the mean, and $\sigma_{x},\;\sigma_{y}$ is the standard deviation.

(4) $$Dissimilarity=\sum_{i,j}\left|i-j\right|P\left(i,j\right)$$

(5) $$Homogeneity=\sum_{i,j}\frac{P_{d}\left(i,j\right)}{1+|i-j|}$$

**Figure 4:**
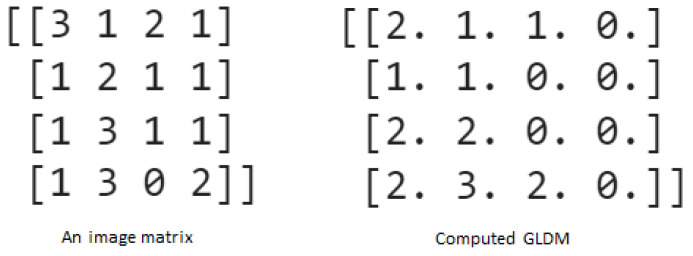
Example of constructing GLDM matrix.

GLDM is used to quantify the relationship between the gray-level values of pixels in an image. A GLDM is a square matrix where each element represents the number of times that a particular combination of gray-level values occurs at a specific distance and direction in the image. Specifically, each element in the GLDM represents the frequency of a pair of gray-level values occurring at a certain distance and direction within the image. The matrix is typically normalized by the total number of pairs of pixels to yield a matrix of probabilities, which are often used as features for texture classification and segmentation. An example of extracting the GLDM matrix from an image is shown in Figure 4, with a distance of one and a direction of 0°.

The wavelet transform is a powerful mathematical tool utilized for signal and image analysis, similar to Fourier analysis. However, in contrast to Fourier transform, which employs sine and cosine functions, the wavelet transform employs wavelets, which are brief and localized in time and frequency. This enables the wavelet transform to analyze signals in both the time and frequency domains simultaneously, capturing both the frequency content and the time-varying behavior of the signal. This makes it particularly useful for analyzing signals with non-stationary characteristics, where the frequency content varies over time. The wavelet transform of a signal f(x) is represented by the following expression:

(6) $$Wf\left(a,x\right)=\frac{1}{a}\Psi \left(\frac{x-b}{a}\right)$$

where *a* is the scale factor and *b* is the translation factor. Since the image is planar, the one-dimensional wavelet transform should be extended to a two-dimensional wavelet transform. For a two-dimensional function f(x,y), the wavelet transform is

(7) $$W_{s}f\left(x,y\right)=f\left(x,y\right)*\Psi_{a,b}\left(x,y\right)$$

where ∗ expresses the convolution along the different directions and *s* is the scale. In two-dimensional images, the intensity of edges can be enhanced in each one-dimensional image by convolving. If the window of the images is convolved in the *x* direction over an image, a peak will result at positions where an edge is aligned with the *y* direction.

The next step in our research methodology was to extract texture features using GLCM and wavelet transforms. Since GLCM and wavelet produce numerous features, we employed a feature reduction technique, namely Fischer score. Following the extraction of texture features, they were incorporated into the deep learning model for subsequent analysis and evaluation.

### 3.3. Deep Learning Model Architecture

**Figure 5:**
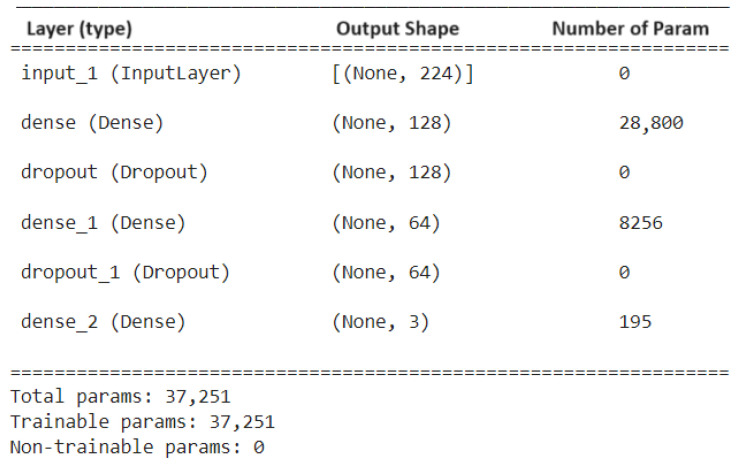
Implemented Model.

The DLNN model depicted in Figure 5 was meticulously crafted to balance performance and computational efficiency. This involved employing a combination of techniques such as pruning, quantization, and compression. Pruning, implemented as a key strategy using weight pruning, involved selectively removing individual weights that were close to zero or had minimal impact on the network’s output. This weight-based approach aimed to reduce the neural network’s overall size and complexity. By eliminating these less important weights, the network became more efficient without sacrificing its performance on classification tasks, ensuring that essential features were preserved for accurate image categorization.

To optimize the model for deployment on resource-constrained devices, careful consideration was given to factors like model size, inference speed, and accuracy. The architecture consists of four dense layers, each followed by a dropout layer to prevent overfitting. Dropout layers randomly deactivate a fraction of neurons during training, enhancing the model’s generalization capability.

The hyperparameters chosen for training the model, as outlined in Table 2, were carefully selected to optimize the training process. The optimizer employed was Adam, a widely-used optimization algorithm recognized for its efficiency and effectiveness in training deep neural networks. To mitigate overfitting, a dropout rate of 0.4 was applied to the dropout layers, serving as a regularization technique. Additionally, the activation function used was softplus, ensuring a smooth and continuous output from the model.

The model was trained for 100 epochs with a batch size of 15 samples per iteration, striking a balance between computational efficiency and stability. To promote stable convergence and prevent overshooting of the minima, a low learning rate of 0.00001 was maintained throughout the training process. Furthermore, cross-validation was implemented to evaluate the model’s performance robustly and ensure its generalizability. These hyperparameters were meticulously chosen based on empirical observations and best practices to guarantee optimal model performance.

By meticulously fine-tuning these hyperparameters and architectural choices, the DLNN model was optimized to achieve superior performance in accurately categorizing chest X-ray images into normal, COVID-19, and viral pneumonia classes, as demonstrated in the results section.

## 4. Results

In this section, we present the results obtained from our experiments, preceded by a discussion on the training process. Our deep learning model was trained on a dataset comprising labeled COVID-19 and non-COVID-19 images, utilizing a standard training-test split. During training, batches of images were iteratively fed into the model, with its parameters adjusted using the Adam optimizer and a predefined learning rate established during hyperparameter tuning. Dropout regularization was implemented to address overfitting by randomly dropping units within the network. In the test phase, the model’s performance was periodically assessed on a separate test dataset to evaluate its generalization ability, utilizing performance metrics such as accuracy, precision, recall, and F1-score. To combat overfitting, early stopping criteria were enacted, ceasing training if test performance stagnated. Additionally, we monitored training and test loss curves, adjusting hyperparameters if overfitting indicators arose. These measures ensured the effective training and reliable performance of our deep learning model for COVID-19 detection. Furthermore, we employed accuracy as the primary metric for initial model assessment, providing an overall measure of correctness in classification. Additionally, to delve deeper into performance evaluation, we utilized recall, precision, and F1-score metrics, offering a more comprehensive understanding of the models’ capabilities in accurately identifying relevant instances and classifying positive instances. Specifically:

- Recall, also known as true positive rate or sensitivity, measures the model’s ability to correctly identify all relevant instances of a class, presenting the ratio of true positive predictions to the total number of actual positive instances, and is given by
  $$\mathrm{Recall}=\frac{\mathrm{True}\;\mathrm{Positives}}{\mathrm{True}\;\mathrm{Positives}+\mathrm{False}\;\mathrm{Negatives}}$$
- Precision evaluates the model’s accuracy in classifying positive instances among all instances predicted as positive, quantifying the ratio of true positive predictions to the total number of predicted positive instances, and is given by
  $$\mathrm{Precision}=\frac{\mathrm{True}\;\mathrm{Positives}}{\mathrm{True}\;\mathrm{Positives}+\mathrm{False}\;\mathrm{Positives}}$$
- F1-Score, a harmonic mean of precision and recall, provides a balanced assessment of the model’s performance, particularly valuable when dealing with imbalanced datasets, and is given by
  $$F1=2\times \frac{\mathrm{Precision}\times \mathrm{Recall}}{\mathrm{Precision}+\mathrm{Recall}}$$

In addition to these metrics, we visualized the classification outcomes through confusion matrices for each model, illustrating the interplay between true positive, true negative, false positive, and false negative predictions across different class categories. Furthermore, to provide a comprehensive view of the model’s discriminatory capabilities, we presented ROC curves, focusing particularly on the deep neural network model due to its superior performance. These visualizations offer insights into the models’ ability to discriminate between different classes and complement the quantitative metrics employed in our evaluation.

**Figure 6:**
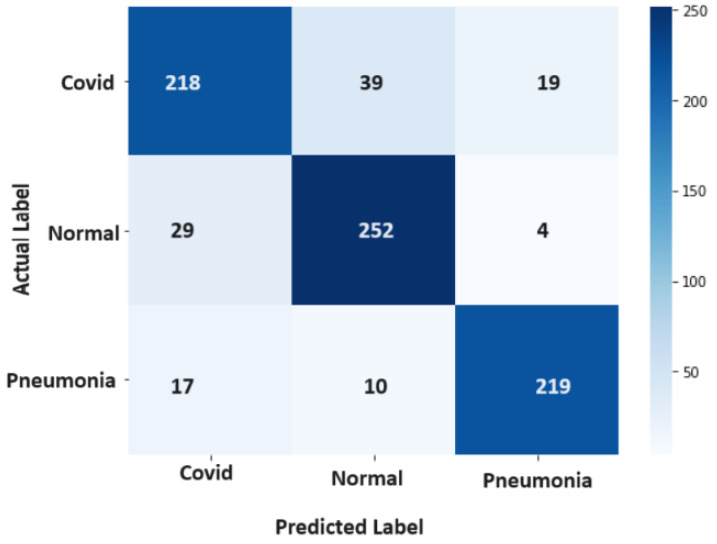
Confusion Matrix-Random Forrest.

**Figure 7:**
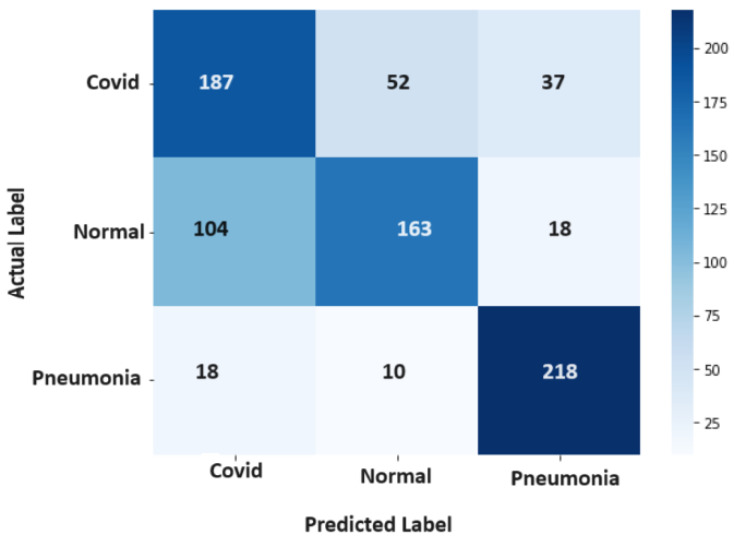
Confusion Matrix-SVM.

Figure 6 provides a visual representation of the confusion matrix stemming from the application of the RF technique, encapsulating the outcomes of RF’s classification process. Similarly, Figure 7 illuminates the confusion matrix originating from the use of the SVM, illustrating the distribution of the SVM’s classifications comprehensively.

**Figure 8:**
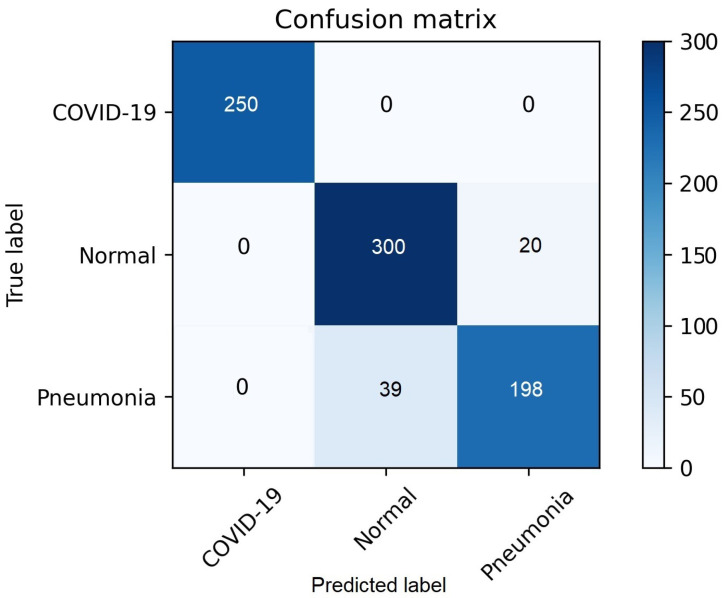
Confusion Matrix-Deep Neural Network.

Expanding on our observations, Figure 8 offers insight into the confusion matrix produced by the deep neural network’s application to the test dataset. This presentation allows for an intuitive understanding of the model’s performance across different class categories. Impressively, our deep learning model showcased remarkable prowess, achieving a categorical accuracy of 92.71%. This achievement, which outshines the accuracy achieved by other employed techniques, reflects the model’s ability to make accurate predictions across the varied classes of chest x-ray images.

**Figure 9:**
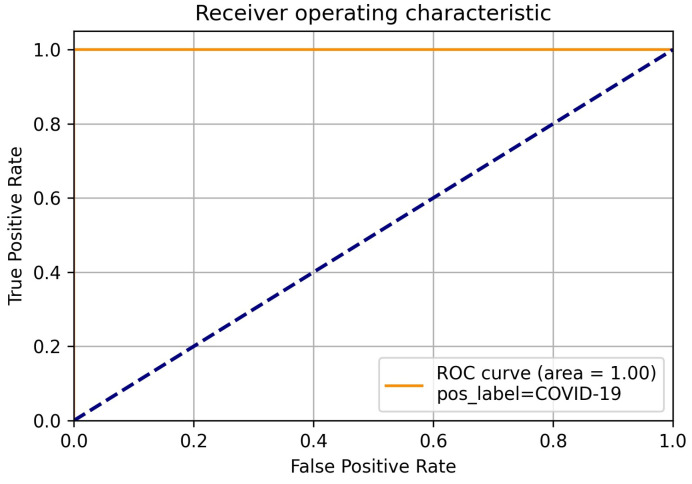
COVID-19 ROC.

**Figure 10:**
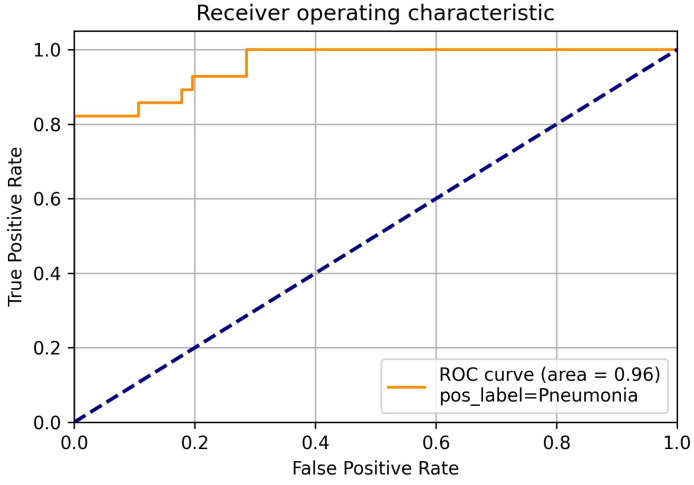
Viral pneumonia ROC.

**Figure 11:**
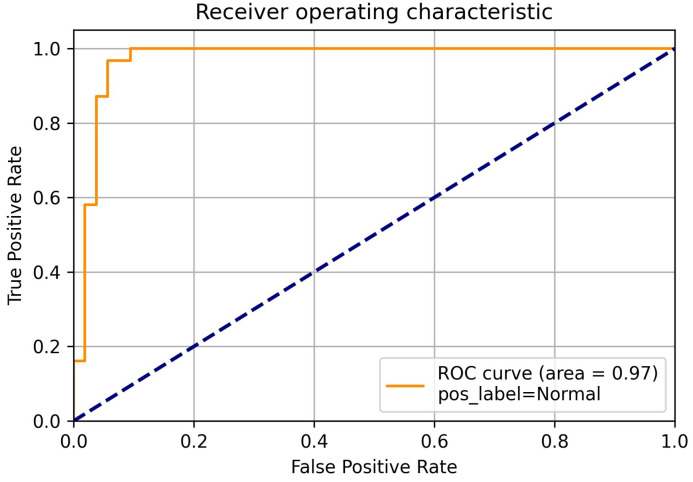
Normal ROC.

Further emphasizing our model’s performance, it’s noteworthy that the RF technique yielded an accuracy of 85%, while the SVM achieved an accuracy of 70%. This stark contrast in accuracy values underscores the distinct strengths and capabilities of each technique in addressing the complexities inherent in the categorization of chest x-ray images. In classification tasks, false positive and true positive values are crucial performance parameters. The ROC curve is generated by plotting false positive values against true positive values. A false positive occurs when the actual value is negative but the prediction is positive. A true positive occurs when the actual value is positive and the predicted value is also positive. Since the DLNN obtained the highest results, we focused on showing the ROC using the DLNN. The ROC curve for COVID-19 is illustrated in Figure 9, while the ROC curve for viral pneumonia is depicted in Figure 10. The ROC curve for normal cases is presented in Figure 11. These curves provide valuable insight into the performance of our model across different classes.

When comparing the performance of three distinct techniques—RF, SVM, and DLNN— across the metrics of recall, precision, and F1-score, discernible trends emerge that shed light on their relative strengths and capabilities, as shown in Table 3.

Starting with the deep neural network’s confusion matrix, the recall values represent the network’s proficiency in correctly identifying instances of each class. Precision values, quantifying the accuracy of positive predictions, were similarly calculated. Further elucidating the balance between precision and recall, F1-score values were computed by harmonizing these two metrics.

Moving on to RF and SVM, it’s evident that these techniques excelled in different aspects. RF attained a recall of 0.85, signifying its ability to accurately capture a substantial proportion of true positive instances. Coupled with a commendable precision of 0.86, RF demonstrated its proficiency in minimizing false positives. Consequently, the F1-score, harmonizing both recall and precision, also reached 0.86.

In the case of SVM, a recall of 0.74 was achieved, indicating its capacity to identify relevant instances within its classes. However, SVM’s precision, measuring its ability to correctly classify positive instances, stood at 0.71. This suggests a higher likelihood of false positives compared to RF and NN. Consequently, SVM’s F1-score was also lower compared to RF and NN, registering at 0.72, illustrating the trade-off between precision and recall that this technique faces.

Overall, the deep neural network exhibited outstanding performance, surpassing other techniques with the highest accuracy, recall, precision, and F1-score values. Specifically, the deep neural network achieved a remarkable recall of 0.93, highlighting its proficiency in correctly identifying instances of each class. Additionally, the precision reached an impressive value of 0.87, indicating the model’s accuracy in classifying positive instances among all predicted positives. Harmonizing both recall and precision, the F1-score attained a commendable value of 0.89, providing a balanced assessment of the model’s performance. This comparative analysis not only underscores the distinct trade-offs associated with each technique but also offers valuable insights into their suitability for accurately categorizing chest x-ray images into normal, COVID-19, and viral pneumonia classes.

In comparison to [2], and as shown in Table 4, utilizing state-of-the-art CNN architectures with Bayesian optimization for COVID-19 diagnosis, our models exhibited competitive performance across various metrics. Notably, our DLNN model achieved an accuracy of 92%, closely rivaling the performance of ResNet50-SVM from the referenced study, which attained an accuracy of 95.23%. Furthermore, while our DLNN model demonstrated a precision of 0.87, the SVM models in the referenced study, such as those employing ResNet18 and ResNet50 architectures, achieved precision scores ranging from 0.90 to 0.95. This suggests that our DLNN model excels at distinguishing true positive predictions from total positive instances, albeit with a slight trade-off in accuracy compared to some models in the literature. Moreover, comparing our results to those presented in [3], we observed that our DLNN model surpassed the NasNetMobile and MobileNetV2 architectures employed in the referenced study. While our model attained an accuracy of 92%, NasNetMobile and MobileNetV2 achieved accuracies of 89.30% and 90.03%, respectively. Additionally, our model demonstrated higher sensitivity and F1-score, indicating its robustness in correctly identifying COVID-19 cases while minimizing false positives.

Additionally, our DLNN exhibited an accuracy of 92%, surpassing Hemdan et al.’s [24] COVIDX-Net and Narin et al.’s [25] Inception-ResNetV2, which achieved accuracies of 90.0% and 87.0%, respectively. Additionally, our model’s accuracy outperformed Wang and Wong’s [26] COVID-Net, which achieved an accuracy of 92.4% in distinguishing COVID-19 cases from non-COVID-19 pneumonia. Furthermore, when compared to Ghoshal and Tucker’s [27] Bayesian CNN approach, which attained an accuracy of almost 90.0% when combined with an experienced radiologist for distinguishing COVID-19 cases from non-COVID-19 viral and bacterial pneumonia, our DLNN model exhibited superior standalone performance. These comparisons underscore the effectiveness of our DLNN approach in accurately identifying COVID-19 cases from other classes, highlighting its potential utility in clinical settings for COVID-19 diagnosis.

Dataset details: Aslan et al. [2] utilized a public dataset consisting of 219 COVID-19 images, 1341 normal, and 1345 viral pneumonia images. Khan et al.’s [3] dataset comprised COVID-19 with 3616 images, lung opacity with 6012 images, normal with 10,192 images, and viral pneumonia with 1345 images. Hemdan et al. [24] utilized a dataset with 25 normal cases and 25 positive COVID-19 images. Narin et al.’s [25] dataset included chest X-ray images of 341 COVID-19 patients. Wang and Wong’s [26] dataset comprised 13,975 CXR images across 13,870 patient cases, with 358 CXR images from 266 COVID-19 patients, 8066 images with no pneumonia, and 5538 images with non-COVID19 pneumonia. Ghoshal and Tucker’s [27] dataset included 68 selected posterior-anterior (PA) X-ray images of lungs depicting COVID-19 cases.

## 5. Discussion

In this study, we developed a novel approach for the classification of chest X-ray images into normal, COVID-19, and viral pneumonia categories using texture-based features extracted from the images. Our methodology involved extracting texture features such as energy, contrast, mean, standard deviation, area, correlation, dissimilarity, and homogeneity using techniques like GLCM, GLDM, and wavelet transform. These features were then utilized in training a DLNN model alongside traditional machine learning algorithms like RF and SVM. Our results demonstrated that the DLNN model outperformed RF and SVM in terms of accuracy, recall, precision, and F1-score, achieving values of 0.92, 0.93, 0.87, and 0.89, respectively. The DLNN model exhibited remarkable capability in accurately classifying chest X-ray images, surpassing the performance of conventional machine learning techniques.

To further contextualize our findings, it is imperative to compare our approach with existing research in the field. A notable study by Aslan et al. [2] achieved accuracy ranging from 95.05% to 96.29% by employing pre-trained CNN models and lung segmentation techniques. However, their reliance on manual lung segmentation introduces subjectivity and potential errors, which our automated approach mitigates. Similarly, Khan et al. [3] achieved accuracies ranging from 93.9% to 96% without lung segmentation but did not address class imbalance, potentially leading to biased results. In contrast, our approach addresses these limitations by automating feature extraction and balancing the dataset, resulting in a more robust and reliable model for COVID-19 diagnosis. Moreover, previous studies [9,10,11,12] have contributed to the field, but they may suffer from limitations such as small dataset sizes, a lack of robust evaluation metrics, or insufficient model explainability. In comparison, our study utilizes a large, well-curated dataset, comprehensive evaluation metrics, and provides insights into the model’s decision-making process. By addressing these limitations and demonstrating superior performance, our approach advances the field of COVID-19 diagnosis using chest X-ray images, paving the way for more reliable and interpretable models that can assist clinicians in accurately identifying and managing COVID-19 cases.

In terms of dataset utilization, our study utilized a comprehensive dataset comprising 10,192 normal, 3616 COVID-19 positive cases, and 1345 viral pneumonia images obtained from a collaborative effort between researchers from Qatar University, Doha, Qatar, and the University of Dhaka, Bangladesh. This dataset diversity ensured the inclusivity of various pathological conditions, enhancing the generalizability of our model. However, it is essential to acknowledge the limitations associated with the dataset, such as class imbalance and potential biases, which could impact the model’s performance.

In addressing the class imbalance issue, we adopted a pragmatic approach by ensuring a balanced representation of each class in our dataset. Specifically, we identified the class with the fewest images and set the sample size for the other classes to match that number. This strategy helped mitigate the imbalance across classes, ensuring that the model was trained on a more equitable distribution of data. By equalizing the class sizes, we aimed to prevent the model from being biased towards the majority class and to promote better generalization performance across all categories. This approach not only enhanced the reliability of our model but also minimized the risk of misclassification and improved the overall robustness of the classification framework.

In conclusion, our study presents a novel and effective approach for the automated classification of chest X-ray images into normal, COVID-19, and viral pneumonia categories using texture-based features and deep learning techniques. By leveraging advanced texture analysis methods and integrating them into a DLNN model, we achieved superior performance compared to traditional machine learning algorithms. Our findings contribute to the ongoing efforts towards developing accurate and efficient diagnostic tools for respiratory diseases, thereby facilitating timely interventions and improving patient outcomes. However, further research is warranted to validate our approach on larger and more diverse datasets and to explore its applicability in clinical settings.

## 6. Conclusions

In conclusion, this study presents a novel approach for the automated classification of chest X-ray images into normal, COVID-19, and viral pneumonia categories using texture-based features and machine learning techniques. The theoretical implications of our research lie in the advancement of medical imaging analysis, where texture features extracted from X-ray images offer valuable insights into the underlying patterns indicative of different pulmonary conditions. Our findings demonstrate the effectiveness of employing deep learning neural networks alongside traditional machine learning algorithms like SVM and RF for accurate disease diagnosis, with the deep neural network exhibiting superior performance metrics in terms of accuracy, recall, precision, and F1-score.

From a practical standpoint, our study contributes to the development of robust diagnostic tools capable of assisting medical professionals in timely and accurate disease detection, especially in the context of the ongoing COVID-19 pandemic. By automating the classification process, our proposed methodology has the potential to enhance the efficiency of healthcare systems, particularly in resource-limited settings where access to specialized expertise may be limited.

However, it is important to acknowledge the limitations of our research. The dataset used in this study, while comprehensive, may not fully capture the diversity of chest X-ray images encountered in real-world clinical settings. Additionally, the generalization of our findings may be limited by factors such as dataset bias and variations in imaging protocols across different healthcare institutions. Further research is warranted to validate our approach using larger and more diverse datasets and to explore the integration of additional clinical variables for improved disease characterization.

## 7. Future Work

Future research in automated disease diagnosis using medical imaging holds significant potential for revolutionizing healthcare delivery. One avenue for advancement lies in integrating multi-modal data, including clinical information and patient demographics, to develop more comprehensive diagnostic models. Additionally, exploring transfer learning techniques can expedite model training and enhance performance, particularly in resource-constrained settings. Furthermore, future research should prioritize longitudinal monitoring and outcome prediction, enabling early intervention and personalized treatment strategies. 

## Figures and Tables

**Table 1:** Dataset split.

	Training Set	Test Set	Total
Normal	1095	250	1345
COVID-19	1025	320	1345
Viral Pneumonia	1108	237	1345

**Table 2:** Model Hyperparameters.

Optimizer	Adam
Dropout	0.4
Activation layer	softplus
Epochs	100
Batch size	15
Learning rate	0.00001

**Table 3:** Performance Comparison of Classification Models.

Metrics	RF	SVM	DLNN
Accuracy	0.85	0.70	0.92
Recall	0.85	0.74	0.93
Precision	0.86	0.71	0.87
F1-Score	0.86	0.72	0.89
AUC/ROC	0.83	0.68	0.91

**Table 4:** Results from the employed methodologies. Datasets: Aslan et al. [2], Khan et al. [3], Hemdan et al. [24], Narin et al. [25], Wang and Wong [26], Ghoshal and Tucker’s [27] dataset.

Authors	Deep Learning Classifier	Accuracy (%)
Aslan et al. [2]	ResNet50-SVM	95.23
Khan et al. [3]	NasNetMobile	89.30
Khan et al. [3]	MobileNetV2	90.03
Hemdan et al. [24]	COVIDX-Net	90.00
Narin et al. [25]	Inception-ResNetV2	87.00
Wang and Wong’s [26]	COVID-Net	92.40
Ghoshal and Tucker’ [27]	Bayesian CNN	∼90.00
Our DLNN Model	DLNN	93

## Data Availability

The dataset used in our study can be accessed via Kaggle website.
